# Motor Control Stabilisation Exercise for Patients with Non-Specific Low Back Pain: A Prospective Meta-Analysis with Multilevel Meta-Regressions on Intervention Effects

**DOI:** 10.3390/jcm9093058

**Published:** 2020-09-22

**Authors:** Daniel Niederer, Tilman Engel, Lutz Vogt, Adamantios Arampatzis, Winfried Banzer, Heidrun Beck, María Moreno Catalá, Michael Brenner-Fliesser, Claas Güthoff, Thore Haag, Alexander Hönning, Ann-Christin Pfeifer, Petra Platen, Marcus Schiltenwolf, Christian Schneider, Katharina Trompeter, Pia-Maria Wippert, Frank Mayer

**Affiliations:** 1 Department of Sports Medicine and Exercise Physiology, Goethe University Frankfurt, 60487 Frankfurt am Main, Germany; l.vogt@sport.uni-frankfurt.de; 2 University Outpatient Clinic, Centre of Sports Medicine, University of Potsdam, 14469 Potsdam, Germany; tiengel@uni-potsdam.de (T.E.); fmayer@uni-potsdam.de (F.M.); 3 Department of Training and Movement Sciences, Humboldt-Universität zu Berlin, 10115 Berlin, Germany; a.arampatzis@hu-berlin.de (A.A.); maria.moreno.catala@hu-berlin.de (M.M.C.); 4 Department of Preventive and Sports Medicine, Institute of Occupational, Social and Environmental Medicine, Goethe University Frankfurt, 60590 Frankfurt am Main, Germany; banzer@med.uni-frankfurt.de; 5 University Hospital Carl Gustav Carus at Technical University Dresden, 01307 Dresden, Germany; Heidrun.Beck@uniklinikum-dresden.de; 6 Sociology of Physical Activity and Health, University of Potsdam, 14469 Potsdam, Germany; fliesser@uni-potsdam.de (M.B.-F.); wippert@uni-potsdam.de (P.-M.W.); 7 Centre for Clinical Research, Department of Trauma and Orthopaedic Surgery, Unfallkrankenhaus Berlin, 12683 Berlin, Germany; Claas.Guethoff@ukb.de (C.G.); Alexander.Hoenning@ukb.de (A.H.); 8 Orthopädiezentrum Theresie, 80339 München, Germany; dr.haag@oz-theresie.de (T.H.); dr.schneider@oz-theresie.de (C.S.); 9 Pain Management, Center of Orthopaedics and Trauma Surgery, Heidelberg University Hospital, 69118 Heidelberg, Germany; Ann-Christin.Pfeifer@med.uni-heidelberg.de (A.-C.P.); Marcus.Schiltenwolf@med.uni-heidelberg.de (M.S.); 10 Department of Sports Medicine and Sports Nutrition, Ruhr-University Bochum, 44801 Bochum, Germany; petra.platen@ruhr-uni-bochum.de (P.P.); Katharina.Trompeter@ruhr-uni-bochum.de (K.T.)

**Keywords:** motor control exercise, stabilization, chronic low back pain, unspecific low back pain, exercise, lumbago, lumbalgia, meta-analysis, nonspecific, LBP, sensorimotor

## Abstract

Low-to-moderate quality meta-analytic evidence shows that motor control stabilisation exercise (MCE) is an effective treatment of non-specific low back pain. A possible approach to overcome the weaknesses of traditional meta-analyses would be that of a prospective meta-analyses. The aim of the present analysis was to generate high-quality evidence to support the view that motor control stabilisation exercises (MCE) lead to a reduction in pain intensity and disability in non-specific low back pain patients when compared to a control group. In this prospective meta-analysis and sensitivity multilevel meta-regression within the MiSpEx-Network, 18 randomized controlled study arms were included. Participants with non-specific low back pain were allocated to an intervention (individualized MCE, 12 weeks) or a control group (no additive exercise intervention). From each study site/arm, outcomes at baseline, 3 weeks, 12 weeks, and 6 months were pooled. The outcomes were current pain (NRS or VAS, 11 points scale), characteristic pain intensity, and subjective disability. A random effects meta-analysis model for continuous outcomes to display standardized mean differences between intervention and control was performed, followed by sensitivity multilevel meta-regressions. Overall, 2391 patients were randomized; 1976 (3 weeks, short-term), 1740 (12 weeks, intermediate), and 1560 (6 months, sustainability) participants were included in the meta-analyses. In the short-term, intermediate and sustainability, moderate-to-high quality evidence indicated that MCE has a larger effect on current pain (SMD = −0.15, −0.15, −0.19), pain intensity (SMD = −0.19, −0.26, −0.26) and disability (SMD = −0.15, −0.27, −0.25) compared with no exercise intervention. Low-quality evidence suggested that those patients with comparably intermediate current pain and older patients may profit the most from MCE. Motor control stabilisation exercise is an effective treatment for non-specific low back pain. Sub-clinical intermediate pain and middle-aged patients may profit the most from this intervention.

## 1. Introduction

Interacting with psychological and social factors, neuromuscular deficits and impairments are a major contributor to the onset, chronification and persistence of non-specific low back pain [1,2,3]. With its effect on reducing biopsychosocial factors’ severity and peculiarity, target-oriented training is crucial in the therapy of chronic nonspecific low back pain [4,5]. Interventions to improve neuromuscular deficits are amongst the most established movement therapy regimes in such low back pain treatment strategies [4,5]. Various meta-analyses have highlighted the effects of sensorimotor movement therapies: motor control [5], stabilisation [6,7] and core-stability [8] exercises all showed superior results than control interventions. These sensorimotor treatments have in common that musculoskeletal control by afferent sensory, in particular proprioceptive, input, central nervous system integration and optimal motor control, to assure functional dynamic joint stability during perturbative situations, are key components [9]. As training principles, appropriate muscle recruitment patterns and timing as the adequate motor answer on perturbations of a (stable) human system are crucial.

More detailed motor control exercises [5] and stabilisation exercises [6] have been shown to elicit superior effects on pain and disability in chronic low back pain when compared to minimal interventions. When compared to other active interventions, motor control and stabilisation exercises provided at least similar [5,6], or even larger [7,8] effects when pain and function are the outcomes of interest [10].

General exercise exerts its effects on low back pain via an analgesic effect; exercise releases beta-endorphins, both spinal and supraspinal, by activating μ-opioid receptors [11]. Following this, an acute, sensible decrease in pain occurs. Exercise and, in particular, motor control stabilisation exercise (MCE), may increase the functional capacity of all involved tissues, leading to a protection against neuromuscular-deficient motor patterns [12].

All things considered, the available meta-analyses and a summarizing network meta-analysis on over 5000 patients with chronic low back pain conclude that sensorimotor training is at least one of the most effective active therapy regimens for non-specific low back pain treatment [10]. Despite these promising findings, the (retrospectively collected) meta analyses conclude that only very low-to-moderate quality evidence is available, with a large between-studies heterogeneity and a large risk of bias [5,10]. A final and prospectively assessed proof for the effectiveness of sensorimotor exercises in the treatment of non-specific low back pain is thus still missing.

A possible approach to overcome most of the weaknesses of a traditional meta-analysis, like unknown publication bias and heterogeneous interventions, is to perform a meta-analysis prospectively [13,14]. In prospective meta-analyses, individual studies are evaluated and determined to be eligible for inclusion before the results of any of the studies are published [15]. This can be afforded by a systematic search in clinical trial registries for planned and ongoing studies following contacts with corresponding authors [16], or, as in the present analysis, by pooling collectively planned ongoing studies from a network, before the results of the individual studies are known [15]. In both, pooled data from concurrently conducted clinical trials are published prospectively. Rigorous meta-analyses, undertaken according to the corresponding principles, was shown to lead to more reliable evidence than a retrospective meta-analysis [13,14].

Exercise effects in low back pain are possibly moderated by environmental factors, such as training characteristics and personal and social factors [17]. To delineate the role of exercise for the treatment of low back pain, a better understanding of the value of these moderating variables is crucial. Well-thought-out, delineation may lead to an evidence-based personalization of such physical activity interventions. Individualizing the sensorimotor therapy with respect to individual skill levels, demands, preferences and potential moderating factors such as pain, physical activity, pain experience, stress and social and medical care environments should lead to a determination of the optimal dose for treating patients as individuals, consequently leading to a greater therapy success [18]. Against the background of the need for high quality evidence on the effectiveness or non-effectiveness and the individualization of motor control stabilisation exercises (MCE) targeting pain and disability in patients with low back pain, the purpose of this prospective meta-analysis was to test the effects of individualized motor control stabilisation interventions in patients with non-specific low back pain. Our hypotheses are that (1) motor control stabilisation exercises lead to a decrease in pain intensity and disability in patients with non-specific low back pain when compared to a control group without additional exercises; and (2) that moderating factors, such as patient and training characteristics, affect the intervention effects.

## 2. Methods

### 2.1. Design

#### 2.1.1. Meta-Level

This analysis adopts a prospective meta-analysis and sensitivity meta-regression design. The patient, study and training characteristics were considered as potential predictors of the pooled effect sizes. The studies and analyses were performed within the MiSpEx Network (Medicine in Spine Exercise Network [19]). The statistical strategy for the meta-analysis has been previously published [20]. We followed the relevant (inter alia Cochrane) recommendations for prospective meta-analyses [13,14,15] when conducting this analysis.

Overall, the results from two major multicentre studies (MCS 1 and MCS 2) with a total of 11 (five and six, respectively) study sites with individual randomisation lists and two smaller, single centre studies (SCS 1 and SCS 2) were pooled. A total of 13 study parts consisting of 18 arms were included in the analyses.

#### 2.1.2. Design at the Study Level

All studies and study site arms adopted randomized controlled designs. The two major multicentre studies (MCS 1 and MCS 2) were, a priori, registered in the German Clinical trials register. The registration numbers are: DRKS00004977 (MCS 1, registration date: 05/16/2013) and DRKS00010129 (MCS 2, registration date: 03/03/2016). The study protocol of the MCS 2 was, likewise, previously published elsewhere [21].

### 2.2. Recruitment, Screening and Inclusion and Exclusion Criteria (Study Level)

Representative volunteers were recruited during clinical low back pain consultation hours, via flyers, local newspapers and bulletins and through personal recruitment. Interested persons were then screened for eligibility. Eligibility criteria were: (1) being male or female aged 18–65 years; (2) chronic non-specific low back pain; and (3) the ability to understand the extent and meaning of the study and to answer a questionnaire without help.

Following the application of inclusion and exclusion criteria, patients were approved by the physician or the approved study investigator in charge. Each participant signed informed consent prior to study enrolment.

### 2.3. Randomization

Participants were allocated to the intervention or control group using a 1:1 (MCS 1, SCS 1 and SCS 2), or 2:1 (MCS 2) ratio, respectively. After the inclusion and completion of the first visit, volunteers were randomized into either the intervention or the control group by an approved study investigator. In the MCS 1, SCS 1 and SCS 2, simple, non-stratified randomization procedures were undertaken; the MCS 2 adopted a block-randomized procedure with n_block_ = 18. All outcome assessors were blinded to the participants’ allocation. Participants were told not to communicate their group allocation to other participants or outcome assessors. The randomization order followed the study inclusion order. Each study site received its own randomization list at the beginning of the study (MCS 1) or once a week from the primary study centre (MCS 2); the randomization sequence was generated using a computer-based algorithm (www.randomization.com).

### 2.4. Outcomes

The dependent outcomes were current pain, chronic characteristic pain intensity and subjective disability. Current pain was assessed using a numeric rating scale (NRS, 11-point Likert scale) or a visual analogue (VAS 0-10 cm) scale. The NRS and VAS values were shown to be almost congruent [22]. Consequently, they were treated as a single outcome measure. Characteristic pain intensity and subjective disability were assessed by the subscale’s characteristic pain intensity (0 = “no pain” to 100 = “the worst pain imaginable”) and disability (0 = “no disability” to 100 = “I was incapable of doing anything”) of the Chronic Pain Grade questionnaire (CPG, 11-point Likert scales, [23]). In contrast to the current pain intensity, the Chronic Pain Grade questionnaire retrospectively assesses the previous three months.

Furthermore, patient characteristics were assessed by the variables: age (years) – sex − body mass index (kg/m^2^). Physical activity was measured by habitual training/exercise volume (minutes per week), type of intervention (MCE vs. MCE + behavioural vs. MCE + perturbation), duration of the intervention (weeks), training frequency (n/week), training duration (minutes/training) and total training volume (treatment) during intervention (1–12 weeks, total minutes).

### 2.5. Effect Estimators

All dependent variables were displayed as means and standard deviations. Standard mean differences (Hedge’s g) between the intervention and comparator effect sizes were calculated based on the mean and standard deviation values for the respective scale and respective visit’s difference to the baseline values. Thus, data for the effects in the short term (3 weeks after randomisation), medium term (12 weeks after randomization) and long term (6 months after randomization) were collected.

### 2.6. Intervention

The interventions were guided (centre-based) and instructed (home-based) by experienced medical training or sports therapists. Following the randomization, intervention group participants undertook a three-week centre-based guided intervention, followed by a nine-week home-based individual training. Intervention participants trained (scheduled) three times (MCS 1, MCS 2, SCS 2) or two times (SCS 1) a week, with a mean duration of 20 min (MCS 2), 30 min (MCS 1), 90 min (SCS 1) or 35 min (SCS2), respectively. The program consisted of four different sensorimotor exercises (all studies). For individualization purposes, all exercises comprised twelve different levels of difficulty. At higher levels, the exercise tasks incorporated additional weights (all studies) and/or were self-initiated, additional, perturbative motor tasks (MCS 2 only) such as ball tapping (single-handed, on the floor or against the wall). Two out of the four exercises directly target the core stabilizing muscles, whilst the two other exercises impacted on motor control indirectly via the upper and/or lower extremities. All exercises were dynamic and are commonly described as (1) quadrupedal/all-fours stability, (2) deadlift/rowing, (3) double leg–single leg heel-pad stance and (4) side planks. Exercises consisted of three series of ten repetitions each. In between series, a two-minute break (self-controlled) was held. An experienced therapist determined the starting level and the level increments based on the axis and plane alignment (extremities, trunk) during motion, as well as on the movement goal (endpoint) accuracy and quality of balance during motion or single movements order. Details on the intervention, its levels and the individualization are provided in the open accessible study protocol [21]. Beyond this standard program, an additional behavioural module intervention was added (MCS 1 and SCS 2). This module and its empirical justification is described in detail elsewhere [24]. Control group participants did not receive any additional intervention from within or from the study centres, but were allowed to proceed with their standard regular care or interventions if they were engaged in one at the time of study inclusion. The intervention within all MiSpEx trials was thus additive; the control group was a classic “control with no additional exercise”.

A standardized training log was completed by the therapist (centre-based phase) and, respectively, by the patient himself/herself (home-based phase), documenting the exercise level and the (if applied) additional weight.

### 2.7. Risk of Bias within Studies

Two of the authors (DN, LV) independently rated the risk of bias of the single study sites; for this purpose, we used the Cochrane Risk of Bias assessment tool II [25]. Following the Cochrane recommendations, bias was rated as outcome-specific and not as study-specific (Cochrane Handbook Version 5.1.0, Chapter 8.7). The outcomes of the studies and, where applicable, the study arms/sites were graded for the risk of bias in each of the following domains: sequence generation, allocation concealment, blinding (participants, personnel and outcome assessment), incomplete outcome data, selective outcome reporting, and other sources of bias. Each item was rated as being at a “high risk”, “low risk” or “unclear risk” of bias. Again, any disagreements were discussed between the two major rater. If a decision was not reached after discussion, a third reviewer (TE) was included to resolve any conflicts.

### 2.8. Measures of Treatment Effects—Main Effects

All participants with data assessments at the respective time point/visit were included in the main effect pooling. For main effects data aggregation, the program “Review Manager 5.3” (RevMan, Version 5.3, Copenhagen: The Nordic Cochrane Centre, The Cochrane Collaboration, 2014) was used. Standardized mean baseline-to-respective visit differences (3 weeks, 12 weeks, and 6 months) and each arm’s sample sizes were used for data pooling. A random-effects meta-analysis model for continuous outcomes was chosen. For variance description, 95% confidence intervals were calculated. Pooled data were displayed using Forest-plots. To test for overall effects, Z-statistics at a 5% alpha error probability level were calculated.

### 2.9. Measures of Treatment Effects–Assessment of Heterogeneity

The effect measures heterogeneity between the study’s results were assessed using the I-squared statistic. An I-squared value greater than 50% is indicative of substantial heterogeneity [25].

### 2.10. Measures of an Interaction of the Treatment Effects–Multilevel Sensitivity Meta-Regressions

To counteract potential heterogeneity, a three-step procedure was undertaken.

First, an omnibus multilevel meta-regression (robust random effects model) was conducted including all (potential) predictors of female proportion risk of BIAS sum score, mean age at baseline (years), habitual training volume (minutes/week), current pain (VAS or NRS), body mass index (kg/m^2^), duration of the intervention (weeks), training frequency (units/week), training duration (minutes/training) and characteristic pain intensity. All effect sizes (n = 51 from 13 studies, 3 week, 12 week and 6 month duration) of all studies/study sites were included. For the multilevel-meta-regression calculations, R (R Foundation for Statistical Computing, Vienna, Austria) was used. The procedure (and R syntax) was followed by the suggestion made in Hedges et al. [26] The study was the random factor nesting term in the model. Tau^2^ estimations were used for between-study variance component determination. Estimator of the covariance matrix of meta-regression coefficients were calculated (including their standard error and 95% confidence intervals).

Second, for non-nested independent between-studies effect size estimates, single level meta-regressions were performed. Potential predictors were the types of intervention (dichotomized, MCE versus other, MCE + behavioural versus other, MCE + perturbation versus other). All 12-week-effect sizes were included as (weighted dependent variable experiments). For the analysis, an SPSS syntax (IBM SPSS 23; IBM, Armonk, NY, USA) was used (David B. Wilson; Meta-Analysis Modified Weighted Multiple Regression; MATRIX procedure Version 2005.05.23). Inverse variance weighted regression models with random intercepts (random effect model, fixed slopes model) with the dependent variables current pain, chronic characteristic pain intensity and disability effect sizes (Hedges’ adjusted gs) were conducted. Two models were calculated.

In case of nonsignificant overall (multilevel or single level) multiple regressions, exploratory meta-regressions were performed for each predictor.

For all meta-regression calculations, data were initially plotted using scatterplot diagrams (for each predictor individually). The type of association between the independent and the dependent variables was visually determined. In case of (1) linear associations, data were processed as real values, (2) curve-linear associations; data were re-calculated using logarithmic transformations (log-association), and, respectively, Taylor-series (u-shaped-associations) to provide linearity for the regression calculation.

Again, *p*-values below 5% were considered significant for all sensitivity analyses.

### 2.11. Risk of Bias Across Studies

Due to the prospective design of the analysis, no risk of bias assessment across all studies/arms (funnel plots/graphs) was performed. All studies and study arms were included.

### 2.12. Effect Estimators’ Level of Evidence

The quality of evidence revealed by the meta-analyses was graded using the tool established by the GRADE working group [27], with the current update developed by the Cochrane working group. Quality evidence was categorized as “very low”, “low”, “moderate” or “high” (plus interim values) as follows: “very low” (the estimate of effect is very uncertain), “low” (further research is likely to change the estimate), “moderate” (further research may change the estimate) or “high” (further research is very unlikely to change the estimate of effect) (plus interim values). The grading starts with the type of evidence (RCT = “high”, observational = “low”, all other study types = “very low”) and is decreased or increased based on study limitations, inconsistencies, uncertainty about directness, imprecise data, reporting bias (decreasing items) or strong associations, dose-response findings and confounder plausibility. Recommendations were derived using the clinical guideline developing tool [28].

## 3. Results

### 3.1. Participants Flow

Overall, n = 2663 persons were screened. Of these, n = 2391 participants were included; 1976 (3 weeks, short-term), 1740 (12 weeks, intermediate) and 1560 (6 months, sustainability/long-term) participants with follow-up visits’ number data assessment (pseudo-intention-to-treat) were analysed.

**Figure 1:**
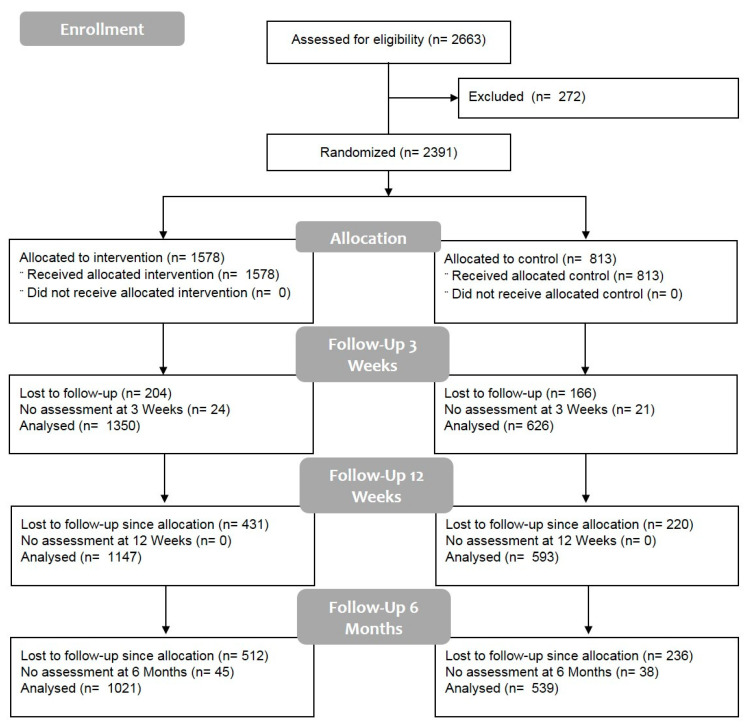
Participants’ flow.

No serious adverse events occurred, however, n = 48 adverse events (AE) did occur. The numbers and reasons for the AE were: 7 musculoskeletal complaints—back-associated, 20 musculoskeletal complaints—other, 4 disease, 5 emotional/psychosocial and 12 other/non-specified. No participant was excluded from one of the trials due to an AE. None of the AEs were undoubtedly effected by the studies’ intervention or outcome assessments. Figure 1 displays the screening procedure and the flow of the participants’ selection and inclusion. All participants for whom outcome measures were available received the allocated condition.

### 3.2. Participants and Study Characteristics

Details of the studies’ and participants’ characteristics are displayed in Table 1. For each of the studies/sites/arms included, methodological aspects, participants’ characteristics and key results are displayed. Between 48% and 65% of the participants in the different studies were female, the mean age ranged between 32 and 49 years and the mean body mass index ranged from 23.3 to 26.2 kg/m^2^. The mean current pain at baseline was 1.5–4.1 points at a VAS/NRS 0-10 scale; characteristic pain intensity (range, 0–100) was 25 up to 50 points. The mean training frequency during week 1 to 12 was (range) 1.7 to 2.7 training units per week.

### 3.3. Risk of Bias Within Studies

The risk of bias ratings for all study and study parts/arm outcomes are displayed in Table 2. As all outcomes are assessed using self-reported Likert scales, they are depicted study-/arm-wise.

### 3.4. Main Effect Estimates

#### 3.4.1. Short-Term Effects

**Figure 2:**
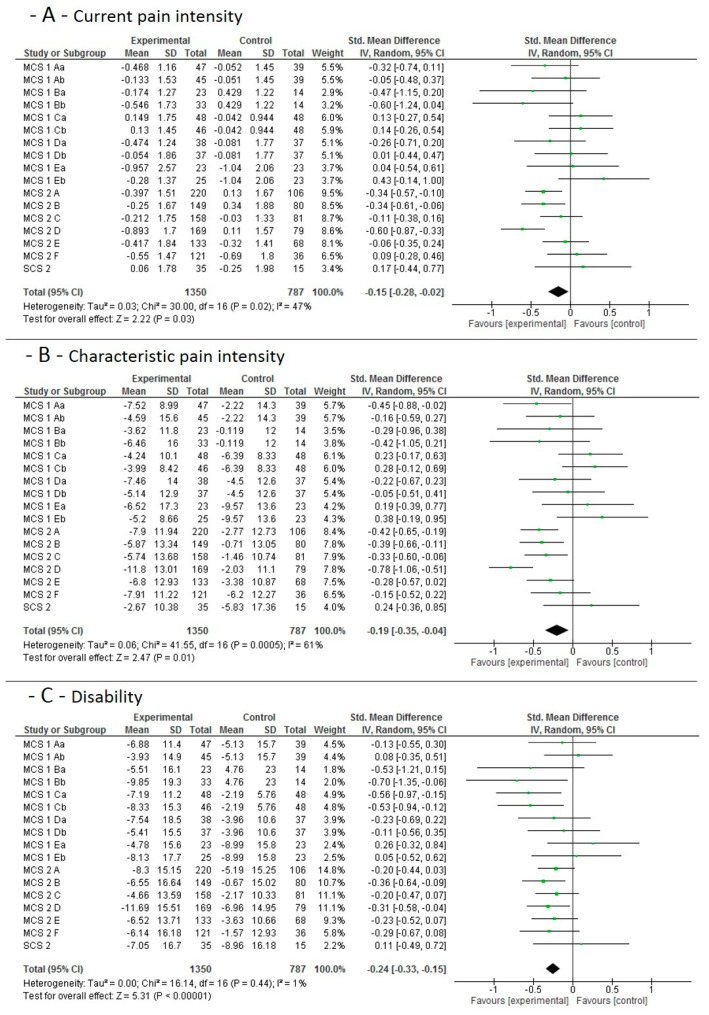
Data and Forest plots for the pooled outcome estimates for the short-term effects of motor control stabilisation exercise vs. no additional exercise. (**A**): current pain intensity; (**B**): characteristic pain intensity; (**C**): disability; MCS, multicenter study; SCS; single-center study; A–F are the single study sites; a stands for MCE, b for MCE + behavioral. SD, standard deviation; P, *p*-value; IV, inverse variance; CI, confidence intervals; experimental, motor control stabilisation group.

The individual studies’ results and the pooled effect sizes for the short-term (3 weeks) effects, when MCE is compared to no additional exercise, is shown in Figure 2. Moderate quality evidence indicates that MCE has a larger effect on current pain and chronic characteristic pain intensity than a control condition without additional exercise. High quality evidence indicates that MCE has a larger effect on subjective disability than a control condition without additional exercise.

#### 3.4.2. Mid-Term Main Study Period Effects

**Figure 3:**
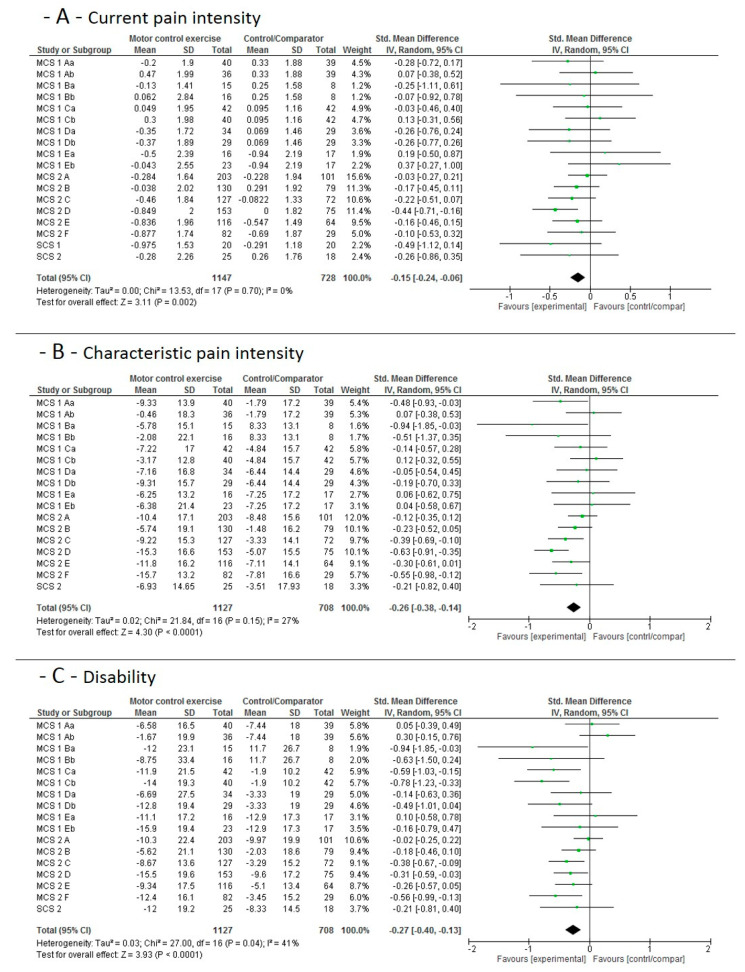
Data and Forest plots for the pooled outcome estimates for the mid-term effects of motor control stabilisation exercise vs. no additional exercise. (**A**): current pain intensity; (**B**): characteristic pain intensity; (**C**): disability; MCS equals multicenter study; SCS equals single-center study, A–F are the single study sites; a stands for MCE, b for MCE + behavioral. SD, standard deviation; P, *p*-value; IV, inverse variance; CI, confidence intervals; experimental, motor control stabilisation group.

The individual studies’ results and the pooled effect sizes for the mid-term (12 weeks) effects, when MCE is compared to no additional exercise, is shown in Figure 3. Moderate-to-high quality evidence indicates that MCE has a larger effect on current pain (moderate), characteristic pain intensity (high) and (high) subjective disability than a control condition without additional exercise.

#### 3.4.3. Long-Term and Sustainability Effects

**Figure 4:**
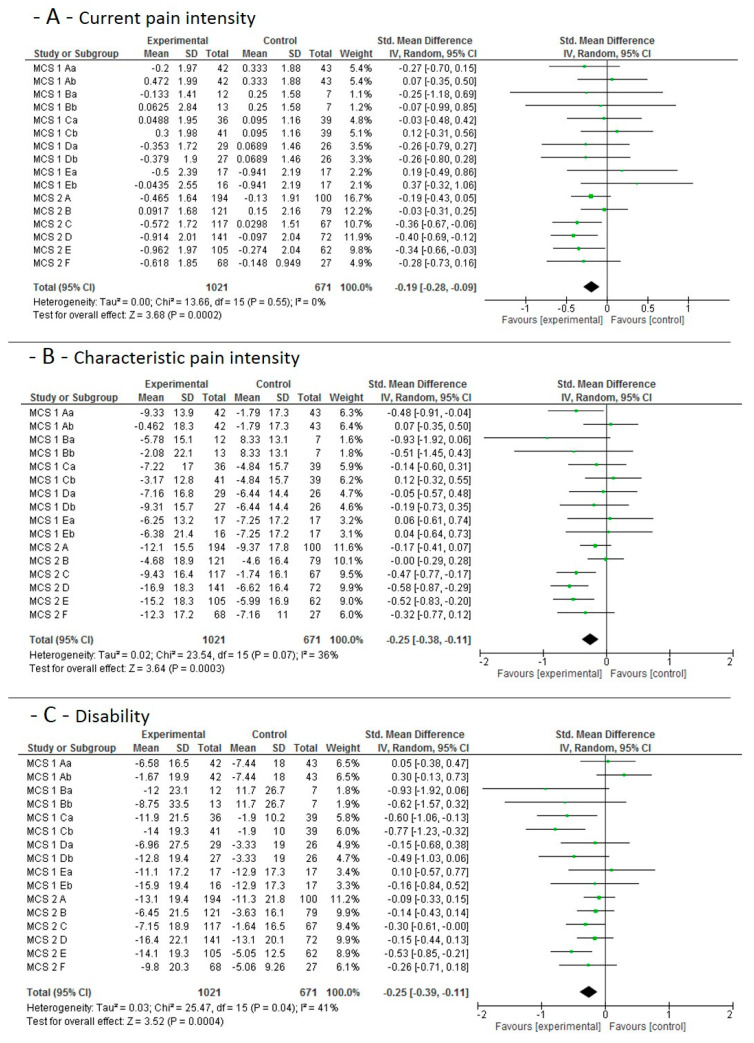
Data and Forest plots for the pooled outcome estimates for the long-term/sustainability effects of motor control stabilisation exercise vs. no additional exercise. (**A**): current pain intensity; (**B**): characteristic pain intensity; (**C**): disability. MCS equals multicenter study; SCS equals single-center study, A–F are the single study sites; a stands for MCE, b for MCE + behavioral. SD, standard deviation; P, *p*-value; IV, inverse variance; CI, confidence intervals; experimental, motor control stabilisation group.

The individual study results and the pooled effect sizes for the long term and sustainability (6 months) effects, when MCE is compared to no additional exercise, is shown in Figure 4. High quality evidence indicates that MCE has a larger effect on current pain intensity than a control condition without additional exercise. Moderate quality evidence indicates that MCE has a larger effect on chronic characteristic pain intensity and subjective disability than a control condition without additional exercise.

A considerable heterogeneity was found. At the main outcome measures for the mid-term effect, heterogeneity was largest for the effects on subjective disability (41%, *p* < 0.05). Subjective disability was thus selected as the dependent variable for the sensitivity analyses.

### 3.5. Sensitivity Analyses

Table 3 displays the characteristics of the sensitivity analyses on the impact of the predictors (independent variables) patient characteristics (who profits the most?) and the training specifics (dose-response relationship) on the MCE vs. control group effect sizes of disability.

No overall (after partialisation of the other independent variables) impact on the predictors on the size of the effect occurred.

**Figure 5:**
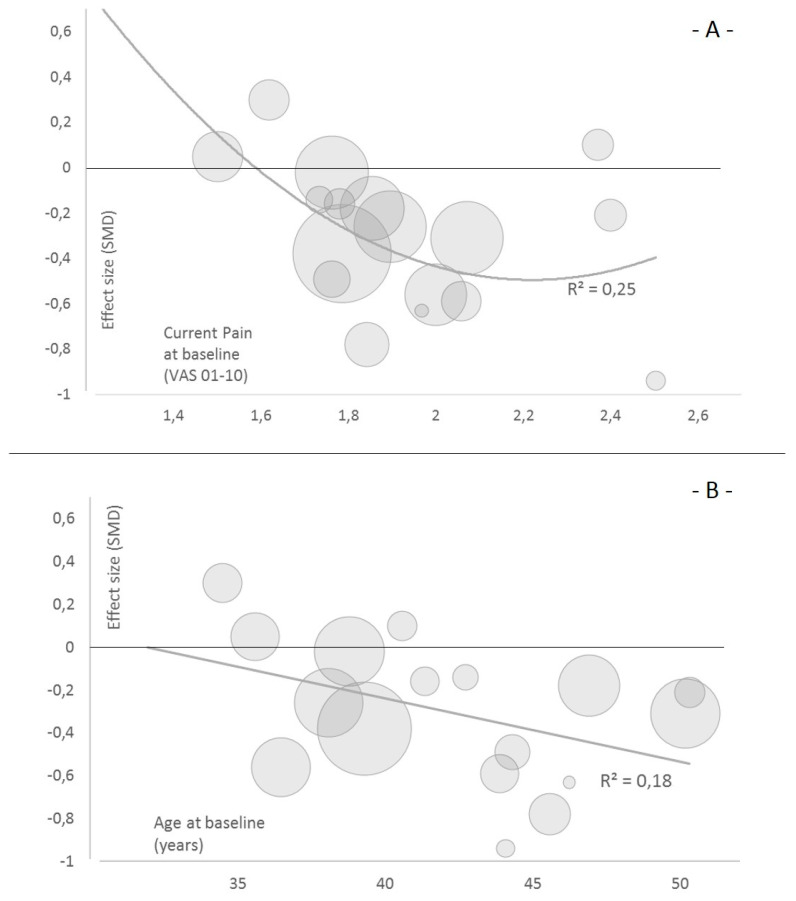
Bubble plots of the meta-regressions (single predictor). (**A**) current pain intensity at baseline and (**B**) participants mean age at baseline on the effect size estimator of the mid-term effect (12 weeks) on disability. The size of the bubble illustrates the weighting of the respective study arm by inverse variance.

Exploratively and at the individual predictor level, current pain intensity explains 25% and age explains 18% of the variance in the effect sizes (*p* < 0.05) (Figure 5). The association of pain and the effect size is U-shaped, whereas a linear (negative) association of baseline age and treatment effect was found.

Low quality evidence suggests that in a low intensity pain sample, such as with intermediate pain intensity of 2 to 2.5 on a 10-point VAS/NRS scale, may profit most. Low quality evidence suggests that in a sample of (on average) 35–50-year-old participants, the older individuals may profit more than the younger ones from an MCE intervention.

## 4. Discussion

### 4.1. Summary of Evidence and Hypothesis Verification

In the short-term, intermediate-term, and sustainability assessments, moderate-to-high quality evidence indicates that MCE has a larger effect on current pain, chronic characteristic pain intensity and subjective disability than a control condition without additional exercise. Low-quality evidence suggests that in a sample of 35–50-year-old participants with mostly low to intermediate pain levels, patients with intermediate pain intensity and older individuals may profit the most from MCE. Hypothesis 1 is thus fully verified, whereas hypothesis 2 is not. Hypothesis 2 can, nevertheless, not be fully rejected.

### 4.2. Comparison with other Evidence

In the high-quality literature that exists, motor-control [5,10] and stabilization exercises [6,10] are found to be superior to minimal intervention on pain and disability decreases. Our findings expand these findings by adding further high-quality evidence to the topic, based on prospective analyses. We found effect sizes (SMDs) of 0.15 to 0.27, corresponding to a small-to-medium effect. When compared to other findings, these effects are rather small. In the short term, effect sizes of (mean) 1.31 are described [10]. In the long-term (sustainability), other authors found long-term effect sizes of 0.5 [8] and 0.44 to 0.46 [30], respectively. The effect size (not the effect itself or the direction) of MCE may have been overestimated in the past; if so, potential reasons lie in the considerable heterogeneity of the effect sizes (26–76% and 86–88%, for example, [5] and [30], respectively). This heterogeneity was recently found to (inter alia) result from differences in the study quality; lower study quality, as found in the recently published network meta analysis [10], has led to larger effect sizes [30].

We included (depending on the analysis), 1560–2391 patients. Beyond the highlighted advantages of a prospective meta-analysis, the number of patients included is comparable, or larger, than the number included in the relevant retrospective meta-analyses. In detail, n = 2431 [5], n = 895 [7,8] and n = 414 [8] partially matching patients were included in these studies.

Rather conflicting results were found with regard to the conducted sensitivity analyses. Low-level evidence for the impact of age and pain on the effect of MCE was found. In other analyses, BMI was not, like in our analysis, a predictor of exercise-based pain and disability changes [31]. Predictors for a lower effect were, however, found in another study, such as being female and higher age [32]. The impact of sex/gender could not be supported by our findings; the direction of the age’s impact on the effect we found was vice versa. Supporting the conclusions, older people benefit 1.27 points more in pain reduction than younger participants after one month of treatment [33]. A negative predictive value of current pain intensity at baseline on therapy success has been described [34]. This seems to be in contrast to our findings. Taking a closer look, severe pain intensity (and not low-to-intermediate pain categorization) predicted a poor treatment response on discharge [35]. Supporting our findings, one may speculate that, on the one hand, patients with moderate pain intensity are able to perform the intervention more accurately due to low pain levels, and, on the other hand, low intensity pain patients leave little room for improvement. On a much higher level of evidence, it seems plausible that high-intensity pain patients need a more multidisciplinary biopsychosocial approach in order to profit [36].

Evidence of the impact of training characteristics on the effect of MCE is sparse. Individually designed exercises have been shown to be effective [35] and to elicit comparably high exercise adherence [35]. The dose-response relationship of MCE in low back pain treatment is yet unknown. Only unpublished material exists at the time of submission of this manuscript. In a meta-regression, a frequency of three to five times per week with a duration of 20–30 min was found to be the most effective (Müller and Niederer, 2020, under review). This supports our scheduled therapy regimen. Furthermore, no unambiguous differences between MCE solely versus MCE plus perturbation versus MCE plus behaviour at the group level for the here-presented sample of persons with mostly low pain intensity was found (when other characteristics of the training are considered in the multiple meta-regression model). In particular, MCE with additional tasks is not more efficient in the general group of low back pain patients than MCE alone [37]. Individualizing the therapy may, nevertheless, result in prescribing only one of the MCE sub-types. The behavioural module may be the most beneficial in patients showing a certain limitation level in pain, distress, social-environment and medical care environment [38].

### 4.3. Practical Relevance

Motor control stabilisation exercise is an effective measure to treat non-specific low back pain in the short-, intermediate- and long-term (sustainability). Yet, only small effects occur at group levels when compared to an inactive comparator. Scheduling MCE three times a week with a duration of 20–30 min leads to a mean training frequency of 1.7–2.7/week. Different training modalities elicited no differences in the achieved effect size. On a group level, sub-clinical but considerable current pain of VAS/NRS 2–2.5/10 may profit the most from MCE, lasting 20–30 min, three times a week. On group level, middle-aged patients (45–50) may profit the most from MCE three times a week, each lasting 20–30 min.

### 4.4. Definition of the Intervention

The intervention adopted is called a “motor control stabilisation exercise”. Historically, motor control exercises are defined as core-specific dynamic stabilization exercises with, mostly, an a priori education on deep trunk muscle activation and/or the control of deep muscle activation during exercising [39]. Here, solely the dynamic/exercise parts were performed. They are often called “coordination”, “stabilisation” [4], “sensorimotor” [21] or even “motor control” [5] exercises. As described above, the term “motor control exercise” may be slightly too specific for the present intervention. On the contrary, “sensorimotor”, “coordination” and “stabilisation” training/exercise may be too general. Consequently, the intervention is named “motor control stabilisation exercise” to highlight that the stabilization/active/dynamic parts of what was originally described as a “motor control exercise” theorem have been adopted. Nevertheless, the intervention can also be named a core-specific stabilization/sensorimotor exercise.

### 4.5. Limitations

#### 4.5.1. Study and Outcome Level

A common limitation in exercise trials is the limited possibility of blinding the participants. The impact of this limitation may be increased by the self-reported assessment of pain and pain-related functions. More importantly, all participants in the MCE groups were not only treated with exercise but also (due to the very nature of the intervention) spent more time with the study personnel and therapists. The impact of this attention to the effect sizes is unknown.

#### 4.5.2. Meta-Level

The transfer of our results to individualized medicine practice may be limited against the low heterogeneity of the training regimen and patient characteristics. A further limitation is given by the fact that the type of intervention could not be included in the overall multilevel sensitivity analysis. The informative value of the non-superiority of one of the intervention types may thus be limited.

At the general level, MCE is effective; future study is warranted to provide high-level experimental evidence on the individualization of the MCE.

## 5. Conclusions

We found moderate-to-high quality evidence for a positive effect of motor control stabilisation exercise on current pain, on chronic characteristic pain intensity and on self-report disability in patients with low back pain when compared to a control without additional exercise. Sensitivity subgroup effects are less straightforward, although it was seen that patients’ characteristics might have an influence. In detail, sub-clinical intermediate pain and middle-aged patients may profit the most. Further high-quality studies are needed to prove or adopt our findings, whilst further evidence is needed to define the individualization criteria on MCE prescription.

## Figures and Tables

**Table 1:** Participants’ characteristics at baseline (all participants) and training (intervention group only) frequency.

Study ID	Females (%)	Age (Years)		Body Mass Index (kg/m^2^)		Baseline Pain Intensity (VAS/NRS 0–10 cm)/Points)		Baseline Pain Intensity (Points 0–100)		Habitual Training/Exercise Volume (Minutes Per Week)		Mean MCE Training Volume (Treatment) during Intervention (1–12 Weeks)	Status in the Publication Process
		Mean	SD	Mean	SD	Mean	SD	Mean	SD	Mean	SD		
MCS 1 A	63	35.3	12.1	23.8	3.79	1.57	1.5	33.2	17.1	242	165	2.0	In preparation
MCS 1 B	65	45.5	8.04	24.6	2.98	2.09	2.5	34.6	23	208	113	1.9	
MCS 1 C	65	37.5	13.3	24.2	4.94	2.06	2.06	39.7	17.6	232	181	2.7	
MCS 1 D	65	41.6	13	24.9	3.56	2.16	1.73	35	20	182	111	2.5	
MCS 1 E	61	38.3	11.6	23.5	2.62	2.43	2.37	35.7	21.6	229	125	2.7	
MCS 2 A	49	38.9	12.4	25.4	3.95	1.65	1.76	25	20.7	2292	125	2.1	In preparation
MCS 2 B	55	46.9	11.9	24.6	3.78	1.64	1.85	29	19.1	276	312	2.0	
MCS 2 C	59	38.6	13.9	24.1	3.5	1.5	1.79	32.4	17.8	198	175	2.7	
MCS 2 D	49	48.1	13,0	26.2	4.91	2.66	2.1	36.2	15	336	321	2.7	
MCS 2 E	65	37.2	11.9	23.6	3.65	1.86	1.9	33.8	20.1	227	207	1.7	
MCS 2 F	56	35.4	13.4	23.5	3.71	1.82	2	35.8	16.1	217	211	2.2	
SCS 1	48	31.8	5.88	23.3	3.9	4.05	1.2	N/A	N/A	N/A	N/A	2.3	Published [29]
SCS 2	56	49.2	13.2	N/A	N/A	4.1	2.4	50.4	16.5	N/A	N/A	N/A	In preparation

MCS, multicenter study; A–F, study sites; SCS, single center study; N/A, not applicable; VAS, visual analogue scale; MCE, motor control stabilisation exercise; SD, standard deviation.

**Table 2:** Individual studies’ outcomes risk of bias.

Study	Selection Bias: Random Sequence Generation	Selection Bias: Allocation Concealment	Performance Bias: Blinding of Participants and Personnel	Detection Bias: Blinding of Outcome Assessment	Attrition Bias: Incomplete Outcome Data	Reporting Bias: Selective Reporting	Other Bias
MCS 1 A	low	high	high	high	low	low	low
MCS 1 B	low	high	high	high	high	low	low
MCS 1 C	high	high	high	high	low	low	low
MCS 1 D	low	high	high	high	high	low	low
MCS 1 E	low	high	high	high	high	low	low
MCS 2 A	low	unknown	high	low	low	low	low
MCS 2 B	low	unknown	high	low	low	low	low
MCS 2 C	low	unknown	high	low	low	low	low
MCS 2 D	low	unknown	high	low	low	low	low
MCS 2 E	low	unknown	high	low	low	low	low
MCS 2 F	low	unknown	high	low	high	low	low
SCS 1	unknown	unknown	high	high	low	low	low
SCS 2	low	unknown	high	high	high	low	low

MCS, multicenter study; A–F, study sites; SCS, single center study.

**Table 3:** Sensitivity meta-regression analyses outcomes, dependent variable = standardized mean differences (Hedges d) of the subjective disability score. Model 1-multilevel meta-regression; Model 2-single level meta-regression on intervention type. Each time, the total effect model descriptives and the contribution of the single predictors (independent variables) are depicted.

**Model 1—Multilevel Meta-Regression**					
N effect sizes included			Tau-square		
51			0.036		
Predictor/Moderator	Estimate	Standard Error	95% Confidence interval		
			Lower level	Upper level	significance
Duration of the intervention (weeks)	−0.01	0.02	−0.04	0.02	n.s.
Intervention frequency (units/week)	−0.78	1.71	−4.14	2.59	n.s.
Habitual training volume (minutes/week)	0.03	0.07	−0.11	0.16	n.s.
Intervention session duration (minutes/training)	−0.08	0.18	−0.44	0.29	n.s.
Female proportion	−1.27	2.04	−5.27	2.72	n.s.
Risk of BIAS sum score	0.06	0.37	−0.66	0.77	n.s.
Mean age at baseline (years)	−0.01	0.04	−0.08	0.06	n.s.
Current pain (VAS or NRS)	−0.95	0.81	−2.54	0.63	n.s.
Body mass index (kg/m2)	0.05	0.23	−0.4	0.5	n.s.
Characteristic pain intensity (0–100)	0	0.03	−0.06	0.07	n.s.
**Model 2—Single Level Meta-Regression**					
	Mean effect size	N effect sizes included	R-square	Homogeneity Q	Homogeneity *p*-value
Descriptives	−0.2772	17	0.0252	0.0077	>0.05
Independent variable	B	Standard error	95% CI	Z-value	*p*-value
MCE versus other	0.211	3.16	−6.3; 6.1	−0.035	>0.05
MCE + behavioral versus other	−0.109	3.15	−4.03; 3.55	0.0067	>0.05
MCE + perturbation versus other	−0.0782	2.97	−5.9; 5.74	−0.026	>0.05

B, partial regression coefficient; CI, confidence interval; n, number; MCE, motor control stabilisation exercise.
